# *Candida glabrata*, Friend and Foe

**DOI:** 10.3390/jof1020277

**Published:** 2015-09-16

**Authors:** Phyllix Tam, Kirsten Gee, Miryam Piechocinski, Ian Macreadie

**Affiliations:** School of Applied Sciences, RMIT University, Bundoora, Victoria 3083, Australia; E-Mails: s3283977@student.rmit.edu.au (P.T.); s3330437@student.rmit.edu.au (K.G.); s3345985@student.rmit.edu.au (M.P.)

**Keywords:** antifungal resistance, azole, candidiasis, ergosterol, opportunistic pathogen, starter culture, statins, Torulopsis, yeast

## Abstract

*Candida glabrata* is mostly good, but, at times, it is an opportunistic pathogen. Previously known as *Torulopsis glabrata*, it enjoyed a good reputation and was even present in starter cultures. Its haploid genome and lack of mating made it an attractive challenge for yeast genetics studies. However, more recently it has become better known due to its character as an emerging cause of candidiasis, and for its resistance to multidrugs that are employed for candidiasis treatment. While now classified as *Candida glabrata*, it is still not a good fit and tends to stand alone as a very unique yeast. In terms of sequence, it is dissimilar to other *Candida* yeast and most similar to *Saccharomyces cerevisiae*.

## 1. Introduction

*Candida glabrata* was previously known as *Torulopsis glabrata*. *Torulopsis* is now an obsolete genera with such yeast being reassigned into other genera including *Cryptococcus* and *Candida* [1]. *C. glabrata* is also found in fermented foods and drinks [2]. Although *Candida glabrata* is recognised as a starter culture, to our knowledge it not used as a pure starter culture. However, it has enjoyed a good reputation, and can lead to ethanol production.

*Candida glabrata* was initially placed in the *Torulopsis* genera because of its lack of hyphae and pseudohyphae formation that defined the *Candida* genus at the time. However, in 1978, it was determined that this was not enough of a distinguishing factor and it was reclassified. In fact, only *C. albicans*, *C. dubliniensis*, and *C. tropicalis* can form hyphae. Belonging to the class Fungi Imperfecti [3], it is also now considered to belong to the *Candida* genus following evidence of its emerging human pathogenicity in *Candida* infections with isolated *C. glabrata* becoming increasingly evident. However, many current reports and reviews still use the name *Torulopsis glabrata* when referring to *C. glabrata*. There are large inconsistencies in the scientific community in the appropriate classification of this species [4].

**Figure 1:**
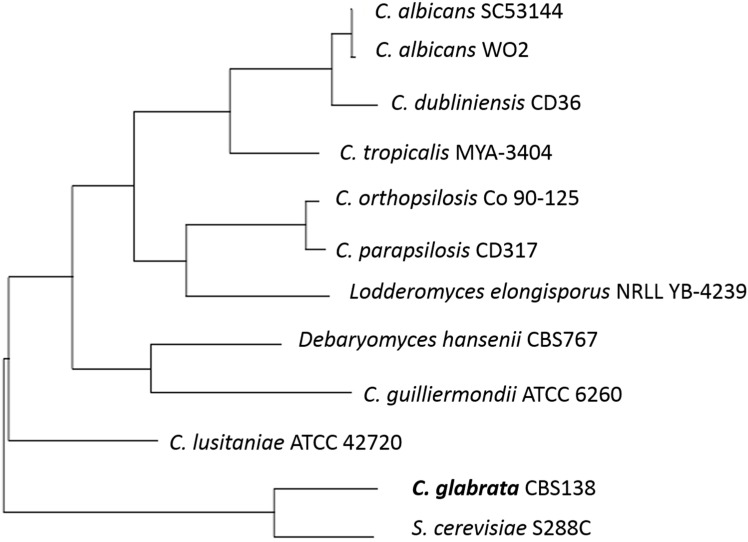
The phylogenetic tree of some yeast species constructed by comparison of *ERG11* sequences and using the tools available at http://www.candidagenome.org/.

Sequence analyses show *C. glabrata* has the highest similarity to *Saccharomyces cerevisiae* (see Figure 1) [5] and much less similarity to *Candida albicans*. As *S. cerevisiae* is primarily known as a food or drink fermenter, similarities to *C. glabrata* should be investigated. It is currently known that the two species of yeast share a relation in important proteins. The Epa proteins of *C. glabrata* that are the main pathogenic adhesins, are closely related to the Flo proteins of *S. cerevisiae*, that are responsible for flocculation in beer fermentation [6]. There is still debate among scientists regarding the biochemistry of flocculation and the process of aggregating cells at the end of the fermentation process through cell wall composition and cell-cell interaction [7]. It could be suggested that there are opportunities within the close protein relationships for further genetic analysis of functions.

Although it is readily found in small numbers on human skin and in mucosa, some evidence of *C. glabrata* in food and beverage fermentation has come to light in various areas. For example, studies have isolated *C. glabrata*, among other yeast species, in traditional ragi in different parts of India using PCR and commercial identification kits to cross analyse and confirm results [8]. Ragi is a grain based food or alcoholic drink starter culture made from finger millet and naturally occurring yeast colonies that have a broad application across domestic India, from porridge, to flatbread and milk substitutes for infants. Despite being isolated and identified in several starter cultures, *C. glabrata* is often found fermenting and coexisting with other species of yeast, particularly *S.* *cerevisiae* and *C. albicans* [9].

*C. glabrata* has also more recently been studied within African fermented food products in relation to assisting the digestion and absorption of minerals in the human gut and its ability to grow in the presence of phytate as a sole phosphate source [9]. Again, this was within a community of related yeast species that shared a similar ability, for example *S. cerevisiae*. Yet another example of African mawe, a local fermented food, looked into the dynamics of spontaneous fermentation and found the predominant yeast identified was *Candida krusei*, followed by *C. glabrata* and *Kluyveromyces marxianus* [9]. Other species found were in lower numbers and considered secondary. Wet processed coffee isolates from another spontaneous fermentation system have also identified *C. glabrata* among some 144 yeasts. These were studied to benefit improvement to the wet process enhancing volatile aroma compounds in coffee beverages using single or multi strain inoculations [10].

Further research is required to investigate why it is always coisolated and if there are situations in food where it may exist as a single isolate. Spontaneous fermentation will generally result in a mix of wild, naturally occurring yeast species that are capable of fermenting in the given environment [11]. Questions should be considered about the possibility of co-dependency of *C. glabrata* and common fermenting yeast species and where this information may lead. It is still obscure whether or not there are benefits to people in using *C. glabrata* in fermentation, however there are certainly new opportunities for deeper research into the functionality of these yeasts in a positive light.

## 2. The Opportunistic Pathogen

*C. glabrata* like most *Candida* species is a part of the normal microbiota of the mouth, gastrointestinal and vaginal tracts in humans [5,12], and, in most individuals, it does not cause disease. However, disturbances in the normal environment appear to lead to *C. glabrata* becoming a cause for disease, especially in immunosuppressed hosts [12,13]. Given the rise of antimicrobial resistance and the limited number of efficient antifungal drug treatments currently available, Rodrigues *et al.* [12] note that *C. glabrata*’s pathogenicity could be attributed to its ability to form biofilms and its relatively high resistance to traditional antifungal therapies. The increased use of immunosuppressive therapies in modern medicine may also contribute to the increased frequency of *C. glabrata* infections.

At present, the virulence factors associated with *C. glabrata*, relative to other pathogenic yeast species like *C. albicans* are poorly understood. When compared with *C. albicans*, *C. glabrata* can sometimes be considered to be “less virulent” [3,5,14,15]. Furthermore, its inability to secrete proteases led it to originally being called *Torulopsis glabrata* [12]. The species was only reclassified to *Candida* because of its human pathogenicity [3]. There is agreement that the two major functional differences between *C. albicans* and *C. glabrata* are the inability of *C. glabrata* to form true hyphae and to secrete certain proteases [16].

To date, there is a lack of understanding surrounding how *C. glabrata* interacts within a host and the host’s defence mechanisms while simultaneously retaining a commensal existence in areas of the body including the mouth, intestines and vagina mucosal surfaces [3,12]. In healthy hosts, *C. glabrata*’s relatively non-pathogenic nature suggests that it has few virulence factors, however, its high mortality rate in immunosuppressed hosts and rapid dissemination suggest otherwise [3]. It is assumed that a non-immunosuppressed host is able to control *C. glabrata* by suppressing the expression of its pathogenic mechanisms but again, little of this has been confirmed [12]. The prevalence of *C. glabrata* infections in cancer patients, transplant recipients and AIDS patients, all of whom have limited T-cell functionality, could indicate that T cells play a role in the protection of *C. glabrata* infections in healthy individuals. However, there are no known reports of increased prevalence of *C. glabrata* infections in B-cell compromised individuals, perhaps indicating that antibodies are not an associated mechanism used to defend the host from *C. glabrata* [3]*.*

By comparing virulence traits, and more importantly, virulence genes in bacterial cells, it has been found that pathogenic strains of bacteria often have chromosomal gene clusters; “pathogenicity islands” that encode for virulence traits. These islands are absent in very similar but non-pathogenic bacterial species [16]. It is hypothesised that *C. glabrata* has evolved in similar fashion, gaining novel virulence traits and associated genes not found in closely related strains *S. cerevisiae* or *C. albicans* [16].

### 2.1. Adherence and Cell Wall

Adherence is considered a prerequisite for tissue invasion and infection. Given that adherence is often the first stage of host colonization, adherence is an extremely important virulence factor. *C. glabrata* can attribute its emerging success as an opportunistic pathogen to the plethora of adhesins present on the cell surface [14,16].

*C. glabrata* has an expansive collection of cell wall organisation genes not found in *S. cerevisiae* [12]. This adaptation could be attributed to *C. glabrata*’s ability to adhere to a variety of surfaces from host tissue to medical devices. *C. glabrata* is one of the most robust *Candida* species and can survive on inanimate surfaces for five months, while *C. albicans* cannot survive beyond four months [17]. Rodrigues *et al.* [12] note that this adaptation has most likely arisen from *C. glabrata*’s response to stresses like oxidative stress, nutrient limitation, competition with other microorganisms and the lack of sporulation. Fidel *et al.* [3] note that *C. glabrata* is not as sensitive to environmental conditions as *C. albicans* despite both species having comparable cell surface hydrophobicity properties. However, in a more recent study it was found that *C. glabrata* has a notably higher relative cell-surface hydrophobicity than other *Candida* species [5]. In fact, *C. glabrata* has about a four-fold greater cell-surface hydrophobicity value than that of *C. albicans* [14]. Fidel *et al.* [3] note that *C. albicans* is better at adherence in a number of environments when compared with other *Candida* species; *C. glabrata* is notably more robust in *in vitro* test conditions. Again, it must be noted that considerably more work has been performed on *C. albicans* compared with *C. glabrata.* Similar to many fungal cells, *C. glabrata*’s cell wall is a point of contact with host cells and can protect the cell from harsh environments, maintain cell shape and perhaps most importantly for *C. glabrata*, it enables adherence [18]. The central core of *C. glabrata*’s cell wall structure is a branched β-(1,3)-, β-(1,6)-glucan linked to chitin by a β-(1,4)-glucan linkage. A number of chitin and glucan chains extend out through the full depth of the cell wall structure. The outermost glycoprotein layer of *C. glabrata* is a key factor in host cell recognition and then adherence [18]. The general structure of this outer layer is comparable to that of *S. cerevisiae*, which has similar mannan sugars decorating these cell wall proteins. It is well documented that glycosylation of these sugars is vital to *C. albicans* virulence; however, little is known as to whether this is the case for *C. glabrata* and its pathogenicity, or whether the species has conserved any glycosylation machinery [18]. A number of glycosylation enzymes are conserved in *C. glabrata* and their deletion reduces virulence [18]. This change in virulence is likely to be due to reduced adherence to host cells, however that has to be further investigated.

The ability of *C. glabrata* to adhere to the epithelial tissue of hosts is mediated mostly by the expression of glycosylphosphatidylinositol (GPI) linked adhesin genes [12,13]. These genes encode the GPI-anchored cell wall proteins that can bind to host cell carbohydrates [13]. Furthermore *C. glabrata* strains carrying particular mutations show an observable improvement in their ability to colonize organs [12]. Two GPI-anchored cell wall proteins, Pwp7p and Aed1p, are not present in either *C. albicans* or *S. cerevisiae*, and their deletion indicates that they are crucial to *C. glabrata*’s adherence [16]. This suggests that *C. glabrata* has evolved unique mechanisms for a more specialised interaction with host cells [16]. However, in comparison to the epithelial adhesin proteins, little more is known about GPI-anchored proteins, making them a promising area of study in the future.

The epithelial adhesin (Epa) gene family encodes a major group of adhesins in *C. glabrata* [12,19]. Studies around the Epa proteins encoded by this gene family are limited. It is known that *EPA1*, *EPA6* and *EPA7* genes do mediate the adherence of *C. glabrata* to human epithelial host cells [13,16,20]. *EPA1*, *EPA4* and *EPA5* make up a cluster of EPA genes located close to a telomere [21]. By studying hyper-adherent mutant strains of *C. glabrata*, Castano and her team [21] confirmed that multiple EPA genes are able to mediate adherence to host epithelial cells and implicates sub-telomeric silencing in the EPA gene regulation, similar to silencing mechanisms observed in *S. cerevisiae*. Despite this, a deletion of *EPA1* alone stops almost all epithelial adherence *in vitro* [21]. A parallel screening of mutant strains of *C. glabrata* found that the deletion of the *EPA1* gene resulted in the deletion of an adhesin molecule, which specifically can recognise host *N*-acetyl lactosamine containing glycol-conjugates [20,21]. The deletion of this highly specific lectin molecule reduced *C. glabrata*’s ability to adhere to human epithelial cells by about 95% [20]. This could be due to the observation that some EPA genes are subject to chromatin based silencing that under normal circumstances represses their transcription, however further study is required [20]. The authors note that *C. glabrata*’s haploid state facilitates genetic analysis as it is much easier to create random mutants and screen for phenotypes of interest when compared with diploid species like *C. albicans* [16,20], unless they are lethal mutations.

An important observation made by West and co-authors [18] is that these adhesion tests observed by Cormack and Castano are based on experiments where the yeast cells remain in prolonged contact with cultured monolayers *in vitro*, whereas *in vivo* this is not an accurate reflection of infection. West and co-authors [18] assessed adherence of *C. glabrata* using flow assays, that more accurately mimic the passing and very brief contact opportunities individual *Candida* cells have with endothelial cells in blood vessels.

Interestingly, a number of studies have shown that *C. glabrata* is much more efficient at invasion of host cells when compared with *C. albicans*. Simultaneous infection with *C. albicans* using reconstituted human oral epithelium cells showed enhanced invasion and increased tissue damage. However whether this is the case in a real host is yet to be determined, there have been no *in vivo* studies surrounding *C. glabrata* infections to date [12]. Conclusions drawn from a recent study of *C. glabrata* cells under hypo-osmotic stress suggest that there is a relation between yeast cell numbers and survival abilities in environmentally stressful conditions. It has been suggested that *C. glabrata* cells in high cell density may be protected by materials released from these cells [22]. Perhaps a similar survival mechanism exists in *C. albicans*; however, further studies will be required.

Bacteria have also been found to further facilitate the adherence of *Candida* species. Oral bacteria are particularly good for yeast growth as they produce an extracellular polymer and increase the environment’s acidity, creating optimal growth conditions for yeast cells [23]. Further characteristics of abiotic surfaces for *C. glabrata* adherence include moderately hydrophobic, and rough surfaces. The roughness of a surface provides the yeast with additional surface area and creates niches that favour adhesion [23].

### 2.2. Biofilm Production

The widely accepted observation that sessile cells capable of successfully producing and secreting biofilms are less susceptible to antimicrobial agents than mobile, planktonic cells also applies to *C. glabrata*’s biofilm production [12]. In fact, *C. glabrata*’s ability to produce biofilms is one of its key virulence mechanisms. The ability of a microorganism to produce this highly structured and coordinated extracellular matrix is associated with a high level of antimicrobial resistance [12,19]. Given that biofilm formation involves cell-substrate and cell-cell interactions, the production of highly specific adhesins is crucial to successful biofilm development [13]. The production of this drug resistant biofilm vastly improves their ability to contribute to human disease but also aids in survival as a commensal organism in a healthy host [19].

Immunological studies have found that the formation of biofilms on denture and oral apparatuses protects *Candida* yeasts from detachment and removal by salivary flow or by physical forces [24]. Studies surrounding *Candida* colonisations on denture surfaces have confirmed that *C. glabrata*’s biofilm formation is promoted by increased serum environments, hence its prevalence in oral cavity related infections [14]. In these studies, it was noted that *C. glabrata* was always present in partnership with *C. albicans*, again suggesting that perhaps *C. glabrata* cannot become pathogenic and cause denture stomatitis without the assistance of its more invasive relative. This however, is yet to be proven. Coco and co-authors [24] go on to suggest that it is *C. albicans*’ ability to form hyphae, a trait not present in *C. glabrata*, which could help *C. glabrata*’s survival on dentures, as hyphal forms can help maintain the structural integrity of biofilms and act as a sanctuary for the notably smaller *C. glabrata* cells that are present.

Interestingly, when compared with other *Candida* strains *C. glabrata* has the lowest biofilm metabolic activity despite having the highest number of biofilm cultivatable cells. Generally, *C. glabrata* biofilms have less total biomass yet higher quantities of proteins and carbohydrates when compared with other *Candida* strains [12]. The multilayer composition of *C. glabrata*’s biofilm includes intimate packing of blastoconidia and the absence of pseudohyphae or hyphae [25].

Until quite recently, it was assumed that *C. glabrata* was incapable of any form of phenotypic switching [14]. In a laboratory study of a particular strain of *C. glabrata*, STE12, when under induced nitrogen starvation this strain was observed to phenotypically switch to a pseudohyphal growth form *in vitro* [26]. The Ste12 transcription factor family involved a number of regulation processes including filamentation and invasive growth, and is highly conserved in many fungal species. In *S. cerevisiae*, Ste12 is one of the major regulators responsible for a morphogenetic switch similar to that observed in the *C. glabrata* STE12 strain *in vitro* [26]. Again, this emphasizes the similarities between *S. cerevisiae* and *C. glabrata*. The study notes that Ste12 plays an important role in *C. glabrata*’s cell wall structure, as when it is deleted the repression of *TIP1* and *CIS3* expression is reduced. These genes code for Tip1 and Cis3, structural components of the cell wall in *C. glabrata* and *S. cerevisiae* [26]. Given the inability for *C. glabrata* STE12 to show filamentation activity *in vivo*, Calcagno *et al.* [26] go on to assume that filamentation is not an important factor in the species’ virulence, and that the many other Ste12 dependent processes could mediate disease initiation in its host, and that these processes are different to those observed in *S. cerevisiae*’s Ste12 protein activities. In contrast to this, Li *et al.* [14] discuss the association of metallotheionin gene (MT-II) and a gene for a haemolysin-like protein (HLP) with *C. glabrata*’s exhibition of phenotypic switching. These phase-specific genes are more similar to mechanisms in *C. albicans*, and these phenotypic switches are easily observed on CuSO_4_ indicator agar plates [14].

*EPA6* encodes the main adhesin involved in biofilm production [13]. It is known that *EPA6* is not expressed *in vitro*, but in the case of *C. glabrata* urinary tract infections *in vivo*, and in particular biofilm growth conditions, *EPA6* is expressed highlighting the species ability to adapt to environmental changes relatively smoothly [12]. Rodrigues *et al.* [12] and Riera *et al.* [13] agree that the complex regulation of these adhesin genes is similar to the regulation of *S. cerevisiae*’s flocculation (*FLO*) genes. This regulation is variable among species and even amongst strains of the same species, however little research has been done in the area of *C. glabrata*’s Epa family of adhesin proteins. High cell density has also proven to induce the up-regulation of the *EPA6* gene, as well as a number of factors. Similar to the regulation of *FLO* genes in *S. cerevisiae*, this multi-factored regulation is proving to represent a major challenge in the pathway of understanding the expression of *C. glabrata*’s *EPA* genes.

Riera and co-authors [13] found a number of molecules and genes, similar to those found in *S. cerevisiae*’s *FLO* genes, in *C. glabrata*’s biofilm formation .e.g., Cst6p transcription factor, Swi/Snf complex and Yak1p kinase/Sir-4 pathway. From their experiments, Riera *et al.* [13] demonstrate that Cst6p is required for *EPA6* regulation and suggest that its role is most likely in chromosome stability and telomere maintenance. There is little information thus far in regards to the exact pathway on how Cst6p regulates *EPA6* expression, but its deletion from *C. glabrata* negatively affects the organism’s ability to produce a viable biofilm [13].

### 2.3. Extracellular Phospholipases and Enzyme Production

Unlike other *Candida* strains, *C. glabrata* does not produce extracellular proteinases/proteases; a hydrolytic enzyme capable of destroying host tissue epithelial cells, and thus vastly improves species’ virulence [3,5,12,14]. However a number of metabolic enzymes produced by *C. glabrata* do not leave this species unarmed in their ability to assault host tissues. It is likely that the species’ pathogenicity is mediated by other hydrolases; like phospholipases [14].

*C. glabrata* produces and releases extracellular phospholipases, heterogeneous enzymes that promote interaction and destruction with host mucus [12,14,27]. Phospholipases can facilitate the infiltration of the epithelial cell’s phospholipid barrier [5,19]. They achieve this by hydrolysing ester linkages in glycerophospholipids, a molecule common in human cell membranes [14]. Phospholipases secreted by *C. glabrata* are the same as two of the phospholipases produced by *C. albicans*, phospholipase B (PLB) and lyso-phospholipase [27]. A review by Ghannoum [27] notes that the association of phospholipase activity and persistent candidemia infections is very strong for *C. glabrata*. The exact mechanisms of phospholipase secretion in *C. glabrata* are not well understood, however, the regulation may be controlled in a similar manner to that which is observed in *C. albicans*. That is, these molecules are released in the early stages of infection, and play a role in the yeast’s ability to adhere, penetrate and then damage host cells [27]. Compared to *C. albicans*, which is known to produce at least four different phospholipases, *C. glabrata* has only been found to produce two, suggesting that phospholipase production is less important to *C. glabrata*’s pathogenicity [14].

*C. glabrata* is also able to produce and release haemolysins, which can then break down blood cells to obtain elemental iron for the yeast’s own metabolic processes. Despite this virulence factor being well observed, the exact genetic expression remains poorly understood at present [12]. However, it has been well documented that the *HLP* gene and relevant products are associated with haemolysis in *C. glabrata* clinical isolates [5].

Similar to *S. cerevisiae*, *C. glabrata* regulates the expression of the catalase gene (*CTA1*) via a complex combination of two catalase regulation genes. The conservation and expression of this gene could provide *C. glabrata* with resistance to peroxide stress. This comes in handy for the species as the production of catalase, an antioxidant defence enzyme, allows *C. glabrata* to resist phagocytosis, a typical host response to host infection [12]. Once phagocytised, fungal pathogens are known to control their metabolism to digest alternative carbon sources, as the internal environment of host phagocytic cells are generally low in glucose sources. Given that C*. glabrata* ferments and assimilates only glucose and trehalose, *C. glabrata*’s autophagy is a key player in its resistance to phagocytosis [3]. Once starved, *C. glabrata* can detoxify reactive oxygen species, causing the disruption of normal phagosomal maturation that leads to phagosome acidification and the inhibition of phagolysosome formation. The induction of this metabolic change is similar to that in *S. cerevisiae* [12].

Adherence, biofilm and phospholipase production are only the major virulence factors discovered thus far in *C. glabrata*. A greater understanding of *C. glabrata*’s virulence factors may provide a new target for therapeutic treatments [27].

## 3. Drug Resistance

Resistance to antifungal treatment by *C. glabrata* was almost unheard of prior to HIV infection [3]. However, with growing numbers of patients being unable to rid invasive candidiasis due to compromised immune systems and/or increased widespread antifungal usage, the drug resistance phenomenon is of immense concern to the medical community. The proportion of azole resistance in clinical isolates across several countries has been shown to increase in the period from 2001 to 2007 [28]. Moreover, a study by Pfaller *et al.* [29] showed resistances to echinocandins of fluconazole-resistant *C. glabrata* isolates was shown to have increased from no cases between 2001 and 2004 to a 9.3% frequency in the time period of 2006–2010 supporting the notion that drug resistance in *C. glabrata* is rapidly developing.

Like all yeast infections, treatment for invasive candidiasis from *C. glabrata* is performed using antifungals. Antifungals provide two effects: fungistatic, whereby the growth of the yeast pathogen is inhibited, or fungicidal where the yeast pathogen is eradicated or killed [12]. Classes of antifungals are categorized based on the drug’s action on the yeast pathogen. Azoles, the most commonly used antifungals, include fluconazole, itraconazole, ketoconazole, voriconazole, posaconazole and rosaconazole, and inhibit the cytochrome P450-dependent enzyme lanosterol-demethylase which is crucial to the biosynthesis of the cell membrane component, ergosterol. The inability of ergosterol to be synthesised results in cell membrane dysfunction such as impaired signalling and transport processes [30].

Polyene antifungals such as amphotericin B, nystatin and primaricin kill fungal organisms by interacting with ergosterol in the cell membrane. This, in turn, creates channels within the cell membrane causing small molecules to leak, resulting in cell death [12]. Although, *C. glabrata* has shown susceptibility to polyenes, its widespread use as an antifungal is uncommon due to its toxic side effects. The more recently introduced antifungals, echinocandins, include capsofungin, micofungin and anidulafungin, and are a typical first line therapy for invasive candidiasis. They function by inhibiting the β-(1,3)-d-glucan synthase enzyme that synthesises the β-(1,3)-d-glucan essential for fungal cell walls [31]. Finally, 5-flucytosine is an antifungal that interferes with the synthesis of proteins. Upon entering the cell through a cytosine permease, the 5-flucytosine is converted into a nucleotide analogue, 5-flurouracil, which is incorporated into RNA subsequently interfering with the synthesis of proteins [31].

As mentioned earlier, antifungal resistance is becoming an increasingly concerning problem hence understanding the mechanisms of resistance is the key to the discovery of new treatment therapies. Resistance can be observed in two ways: primary resistance, whereby the *Candida* species is inherently unaffected by the antifungal without prior exposure; or secondary resistance, where the species acquires resistance following exposure to the drug [3]. Although *C. glabrata* experiences primary resistance to some extent, as illustrated by its inherent low susceptibilities, secondary resistance is by far the more alarming method.

### 3.1. Azole Resistance

**Figure 2:**
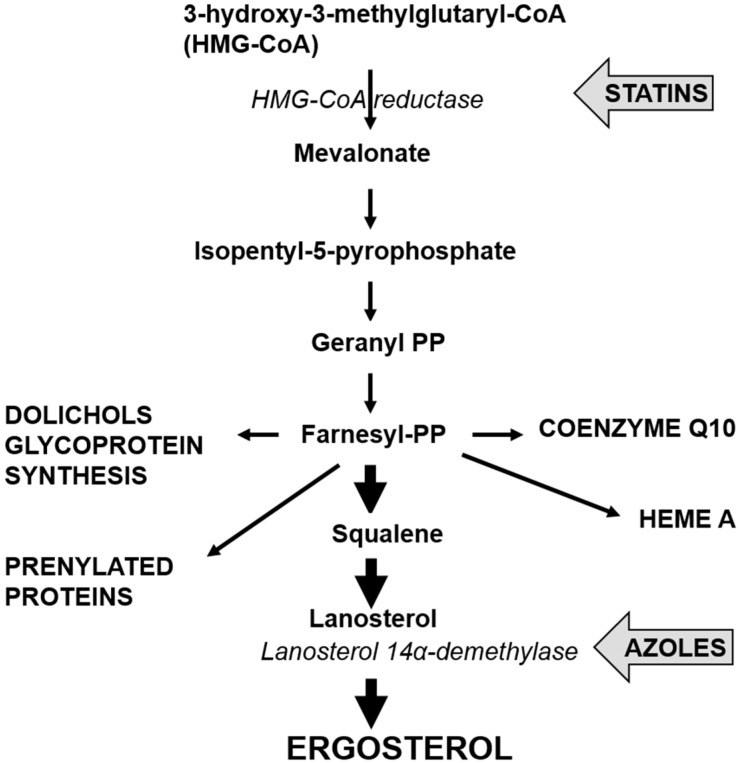
Pathway for the synthesis of ergosterol, showing sites for inhibition by statins and azoles.

Of the *Candida* species, *C. glabrata* appears to have the least sensitivity to azoles [3]. Additionally, the growing use of azole antifungals to treat invasive candidiasis has led to the selection of many resistant strains [28]. The mechanisms responsible for azole antifungal resistance in *C. albicans* are largely agreed to be: (i) changes in the cell wall or plasma membrane leading to reduced drug uptake; (ii) changes in the P-450 lanosterol demethylase enzyme (see Figure 2), encoded by *ERG11*, which results in loss of drug affinity or overexpression of *ERG11*; and (iii) employment of an energy-dependent drug efflux mechanism mediated by membrane transport proteins of the ATP binding cassette (ABC) transporter superfamily or the major facilitator (MDR) superfamily [32]. Of these three mechanisms, the latter two are the most widely studied and have been observed as resistance mechanisms employed by *C. glabrata*.

Genes associated with drug efflux mechanism are *CDR1* and *CDR2* [28,33]. In fluconazole resistant *C. glabrata* isolates, most isolates had up-regulated *CDR1* and *CDR2* [33] with *CDR1* consistently expressed more than *CDR2*. Regulation of *ERG11*, a gene encoding the azole target enzyme (P-450 lanosterol demethylase) has also been associated with azole resistance, with increased *ERG11* expression observed in resistant isolates [34,35]. However, *ERG11* in some azole resistant isolates remained at similar levels to susceptible isolates. [33,35,36]. Therefore, *ERG11* may play a minor role in azole resistance. Interestingly Redding *et al.* [35] also had a resistant isolate that had no measurable overexpression of either *CDR1*, *CDR2*, *SNQ2* or *ERG11* suggesting that an alternate, yet to be identified gene is also responsible for azole-resistant behaviour.

Due to the number of genes established to be associated with azole resistance, it is clear that azole resistance in *C. glabrata* is a complex mechanism that needs further investigation. Moreover, a transcription factor, Pdrp1, regulates the expression of *CDR* genes by binding directly onto the azole drug to activate the expression of the efflux drug genes. In a study conducted by Ferrari *et al.* [28], 57 single point mutations located in 3 main hot spots of the *PDR1* gene were observed to cause hyperactivity to the transcription factor CgPdrp1 resulting in azole resistance. Disruption and/or removal of the *PDR1* gene was found to increase susceptibility to azoles supporting the notion that *PDR1* plays a distinct role in azole resistance in *C. glabrata* isolates. Possible investigation into the inhibition of this transcription factor could prove beneficial in combatting azole resistance. The study also found that the mutations to *PDR1* would enhance virulence and resistance in mice models suggesting the rise of Gain Of Function (GOF) mutations. This characteristic is particularly worrying to the medical community as previously increased antifungal resistance was always correlated to a decrease in yeast pathogenic fitness: however, this study suggests otherwise. Therefore, when treating a patient, we must be wary that an azole resistant *C. glabrata* strain may have an increased virulence *in vivo*. Further investigation into GOF mutations needs to be conducted in order to understand and address this issue.

### 3.2. Echinocandin Resistance

Due to the rising resistance of *C. glabrata* isolates to azole antifungals, different classes of antifungals are being explored to combat invasive candidiasis. It is well documented that echinocandins were the most recent class of antifungals to be used in yeast infection treatment. The drug acts on the yeast pathogen in a different manner to azoles and in the first decade of use the occurrence of cross-resistance between the two antifungals was rare [29]. Furthermore, the drug has a good safety profile and was found to cause lower resistance in a broad range of *Candida* species [37]. With these qualities, it was not surprising that echinocandins were and still are recommended as first-line therapy for invasive candidiasis. However, there is currently an increase in echinocandin resistance [37]. Unlike azole antifungals, secondary resistance to echinocandins has conclusively been unrelated to the drug-efflux mechanism as they prove to be poor substrates for most multidrug efflux transporters [38]. Instead, echinocandin resistance is caused by interference between the drug and the target enzyme β-(1,3)-d-glucan synthase. The target subunits for this enzyme are encoded by three genes, *FKS1*, *2* and *3* and the mutations in gene *FKS1* and *2* have been linked to secondary resistance of echinocandins in *C. glabrata* [39]. Mutations in “hot spots” of *FKS1* and *FKS2* cause a high prevalence of amino acid substitutions conferring echinocandin resistance [40]. Interestingly, all the amino acid substitutions in *FKS2* caused a greater decrease in susceptibility to echinocandins suggesting that mutation in *FKS2* plays a greater role than *FKS1* in echinocandin resistance [40]. The incidence of multi-drug resistance between different classes of antifungals is a major concern and recent studies have detected strains of *C. glabrata* resistant to both fluconazole and echinocandins. In the study by Pfaller *et al.* [29], the mutations observed in isolates with fluconazole resistance were also associated with the *FKS* genes. Interestingly F659Δ and S663F were involved in cross-resistance to echinocandins and fluconazole but F625Δ was not observed in the study. This suggests that each isolate can harbour different mutations and levels of resistance and treatment should be isolate specific.

### 3.3. Polyenes Resistance

Research regarding antifungal resistance in *C. glabrata* is largely focused upon azole and echinocandin treatment. Coupled with the rarity of resistance to polyenes, little information is known about the exact mechanism for resistance to polyene antifungals in *C. glabrata* [41]. As mentioned previously, the main target for polyene antifungals is the vital cell membrane component ergosterol. A clinical isolate susceptible to polyene treatment was observed to have severe changes in the sterol composition of its cell membrane [41]. Instead of having ergosterol in its cell membrane, the resistant isolate had accumulated numerous sterol intermediates that were still able to maintain membrane viability. The study found that a missense mutation in the *ERG6* gene caused an amino acid substitution resulting in a change to the subsequent protein, C-24 sterol methyltransferase. This was reasoned to have caused a change in the biosynthesis pathway of ergosterol. In addition, real time PCR was carried out in this study to investigate the gene expression of multiple genes responsible for controlling the ergosterol biosynthesis pathway. Overexpression of the genes encoding enzymes in the later stage of ergosterol biosynthesis was observed and reasoned to be responsible for the accumulation of other sterols in the cell membrane. A similar study by Vandeputte *et al.* [42] also demonstrated a mutation, nonsense as opposed to missense, in the *ERG6* gene and obtained similar results with altered sterol composition in the cell membrane. Interestingly, it is known that polyene resistance is associated with azole resistance. This is because the lack of ergosterol reduces the importance of the role for the P450-dependent enzyme lanosterol-demethylase: hence reducing azoles will have a lesser effect on the yeast pathogen. Although both isolates were observed to up-regulate the *ERG11*, *CDR1* and *CDR2* genes, suggesting an enhancement of the yeasts ability for azole resistance, the opposite was observed in this study. The exact reasoning for these observations was not elucidated but it was suggested the presence of toxic intermediate sterols play a role. Hence further investigation into this anomaly is required. In addition, as only two isolates have been studied to date for the mechanism of polyene resistance it is recommended further investigation be conducted for this.

## 4. Cholesterol Lowering Statins and *Candida glabrata*

Although not designed as antifungals, statins, the main drugs used for treating hypercholesterolemia have antifungal properties [43]. Two of these, simvastatin and atorvastatin, are the first blockbuster drugs reaching billion dollar sales figures for Merck and Pfizer. Many millions of people take these drugs on a daily basis to inhibit the activity of HMG-CoA reductase (see Figure 2) and reduce cholesterol synthesis to acceptable levels. However, HMG-CoA reductase is also present in all eukaryotes and in *Candida* the statins lead to reductions in ergosterol production that inhibit growth. Their end effect on cells is similar to the azoles: both lead to lower ergosterol levels and that inhibits growth [44].

Not all *Candida* species are equally sensitive to simvastatin and atorvastatin: *Candida glabrata* is less sensitive than *Candida albicans* [43]. This suggests that people taking the statins might reduce candidemia or result in different *Candida* populations to those found in the untreated normal population. Some studies have reported reduced candidemia for statin users [45,46] while another study reported no effect on outcomes [47]. However, it was noted that in the last two years of their study, non *Candida albicans* species, particularly, *C. glabrata* and *C. parapsilosis* increased [47]. This is in agreement with the finding that *C. glabrata* is less susceptible and may emerge to be a bigger problem with high statin use.

It is also noteworthy that statins can be associated with muscle weakness and reduced performance in athletes. After growth with simvastatin, *Candida glabrata* also has reduced performance: specifically, reduced respiratory growth. The reduced performance is due to simvastatin causing a loss of the mitochondrial genome, which is required for respiratory function. The loss of the mitochondrial genome may be related to reduced ergosterol levels in the mitochondrial membrane and reduced activity of the mitochondrial DNA polymerase, which is associated with the mitochondrial membrane. It is plausible that humans also experience reduced mtDNA synthesis while on statins and this could lead to reduced ability for mitochondrial respiration [48].

## 5. Conclusions

*Candida glabrata* appears good for healthy individuals. However, in challenging situations where the natural organisms are not in balance, it can exhibit robust growth and present problems. We can learn much from *Candida glabrata*, and indeed it deserves more attention, since it has an existence in a class all of its own.
